# Immune Monitoring of Paediatric Patients Infected with *Rickettsia rickettsii*, *Ehrlichia canis* and Coinfected

**DOI:** 10.3390/pathogens11111351

**Published:** 2022-11-15

**Authors:** Laura Garcia-Rosales, Angelica Escarcega-Avila, Moises Ramirez-Lopez, Diana Manzanera-Ornelas, Enrique Guevara-Macias, Maribel Vaquera-Arteaga, Carolina Alvarado-Gonzlaez, Blanca Elisa Estrada, Florinda Jimenez-Vega, Luis Donis-Maturano, Gerardo Pavel Espino-Solis

**Affiliations:** 1 Veterinary Sciences Deperment, Autonomous University of Ciudad Juarez, Ciudad Juarez 32310, Mexico; 2 Children’s Specialized Hospital of Chihuahua, Av. Carlos Pacheco Villa s/n, Cerro Coronel. II, Robinson, Chihuahua 31090, Mexico; 3 Facultad de Estudios Superiores Iztacala, Universidad Nacional Autónoma de México, Tlalnepantla de Baz 54090, Mexico; 4 Laboratorio Nacional de Citometría de Flujo, Facultad de Medicina, Autonomous University of Chihuahua, Circuito Universitario s/n, Campus II, Chihuahua 31125, Mexico; 5 Traslational Research Laboratory, Facultad de Medicina, Autonomous University of Chihuahua, Circuito Universitario s/n, Campus II, Chihuahua 31125, Mexico

**Keywords:** coninfections, chemokines, *Ehrlichia canis*, flowcytometry, *Rickettsia rickettsia*, T cells

## Abstract

In 2021, 273 Rocky Mountain spotted fever cases were reported nationwide in Mexico. In Chihuahua City, fourteen samples were obtained from children suspected of rickettsial infection. The analysis of samples (January to December 2021) showed prevalence rates of 28.5%, 43%, and 28.5% for *Rickettsia rickettsii*, *Ehrlichia canis*, and both pathogens in coinfection, respectively. The analysis of clinical haematological and biochemistry analytes showed alterations; 100% of the children had elevated liver enzymes and coagulation times, 64% showed leukocytosis due to neutrophilia, 55% had thrombocytopenia, lymphopenia, and hypoalbuminemia, and 45% showed normocytic normochromic anaemia. Statistically significant differences were observed in the expression of the chemokines IL-8, RANTES, CXCL9/MIG, and CXCL10/IP-10 across the coinfected and control groups, and the difference in IP-10 expression was significant for patients infected by *R. rickettsii* compared to the control group. Additionally, significant differences were observed for expression levels of IL-1β, IL-6, IL-17, IFNγ, and TNFα among the *R. rickettsii*-positive group compared to the control group. On the other hand, the coinfected group exhibited modified levels of IL-6, IL-8, and IL-10 compared with the control group. Finally, significant differences were observed for CD8^+^ T lymphocyte subpopulations between individuals positive for *R. rickettsii* and those positive for *E. canis.*

## 1. Introduction

Tick-borne diseases are important public health issues from clinical and veterinary perspectives in Mexico, especially the often-neglected Rocky Mountain Spotted Fever (RMSF) caused by the bacteria *R. rickettsii*, with 273 tick-borne cases reported nationwide in Mexico in 2021 [1]. The State of Chihuahua reported 76 confirmed cases, of which 59 were caused by RMSF and 17 by another rickettsiosis not identified. This number of cases has pushed this state into second place of increasing clinical cases reported in Mexico from 2020 to 2021 [1]. These tick-borne diseases are responsible for high lethality in infants, and require immediate monitoring [2].

Rickettsiosis has been recognized since 1930 in the northern border states of Mexico. From 1985 until the present, these ailments were responsible for outbreaks in Sinaloa, Sonora, Durango, Nuevo León, Coahuila, and the South-eastern states of the USA [3,4]. Different research groups reported a return of rickettsiosis in the first years of the 21st century, with lethality ranging from 20% to 70% [5,6,7,8]. Likewise, rickettsiosis is classified as an emerging disease of public health importance in Mexico, where thousands of cases and hundreds of deaths have been reported in humans [9,10] due to the difficulty of its diagnosis and fatal outcomes when there is no timely intervention in treatment. Furthermore, it is considered an occupational risk disease [5] and is important for travellers, especially in states bordering northern Mexico [6]. Rickettsial diseases are caused by intracellular bacteria belonging to the rickettsial order, such as *R. rickettsii*, *E. canis*, and *Anaplasma phagocytophilum* [7].

In northern Mexico, these infections are classified as zoonotic vector-borne diseases. The vector *Rhipicephalus sanguineus*, also known as the brown dog tick, has a cosmopolitan distribution and is frequently found in the peridomestic environment, causing massive infestations as a consequence of its introduction by dogs [8,11]. Several bacteria, viruses, and protozoa are transmitted by ticks, but bacteria are responsible for the vast majority of the reported tick-transmitted infections [12,13].

The clinical manifestations of rickettsial disease range from a febrile and nonspecific syndrome with temperatures >40 °C that progress to multisystemic organ failure and death if it is not diagnosed and treated in time. Patients may also present with other unspecific symptoms, such as vomiting, nausea, headache, myalgia, arthralgia, and more. Consequently, these illnesses are usually confused with other common pathologies in children [14]. These ailments may cause various respiratory, gastrointestinal, nervous, and other clinical symptoms [7]. Additionally, they are characterized by the stimulation of an inflammatory response that is reflected in the clinical alterations observed in the acute phase of infection [15], and alterations, such as anaemia, thrombocytopenia, lymphocytosis, leucopenia, elevated liver enzymes, coagulation times (prothrombin and thromboplastin), hypoalbuminemia, and hypoproteinemia, may be observed [16]. These clinical signs are associated with the expression and secretion of different proinflammatory mediators [13] and are sometimes potentiated by the presence of other infectious agents in coinfection [17]. Coinfections aggravate clinical signs, complicate diagnosis, and reduce the time frame for opportune, correct, and specific diagnosis and treatment [18].

In terms of paediatric cases of Rickettsiosis caused by *R. rickettsii*, Martínez-Medina et al., 2005 recorded two paediatric cases in Sonora [19]. Leon–Arias et al., 2008 reported eight confirmed cases of Rocky Mountain Spotted Fever (RMSF), in which 75% of the patients were infants and 50% of the cases ended in fatality [20]. In a 9-year retrospective study conducted by Gómez-Rivera et al., 2013, at Sonora State Children’s Hospital, they recorded a total of 116 children considered as probable positive, of which only 86 (74.6%) were confirmed. Moreover, a cross-sectional study of 47 patients who died of RMSF reported that over three years in the state of Sonora, 49% of affected patients were infants [21].

In terms of coinfections, coinfection of Dengue virus and *R. rickettsii* was reported in one woman, and coinfection of *R. rickettsii* and *Leptospira* spp. was reported in a 12-year-old boy who presented nonspecific signs that rapidly progressed to gastrointestinal, hepatic, renal, and neurological dysfunction [22].

The pathogenesis of the disease starts when Rickettsia invades endothelial cells, promoting their dissemination and stimulating cell signalling cascades, which leads to cytokine secretion, infiltration of macrophages, CD4^+^ and CD8^+^ T lymphocytes, and NK (natural killer) cells into the vascular cell wall and perivascular space [23]. As a result of invasion, the endothelium acquires an activated inflammatory phenotype [24]. Human cell in vitro models show that endothelial cells increase the expression and secretion of cytokines interleukin-1 (IL-1), IL-6, and tumour necrosis factor α (TNFα). Chemokines such as CXCL8/interleukin-8 (IL-8), CXCL10/interferon gamma-induced protein 10 (IP-10), CCL2/monocyte chemoattractant protein 1 (MCP-1), CXCL9/interferon gamma-induced monokine (MIG), and CCL5/RANTES lead to the activation and recruitment of leukocytes to the site of infection, resulting in the potentiation of the inflammatory response and its termination [23,25,26,27,28]. Once infected, monocytes, immature macrophages that circulate in the bloodstream, promote the spread the pathogens in the vascular endothelium, causing an increase in microvascular permeability, which is the main pathophysiologic effect in this disease [29,30,31,32].

The defence mechanisms against pathogenic rickettsial bacteria in humans are quite complex and poorly understood; nonetheless, there is a proposed mechanism supported by evidence from animal models and cell cultures [24]. The immune mechanisms by which the host kills and controls rickettsial bacteria are highly dependent on cellular immunity, with a critical role identified for T lymphocytes [33,34]. Animal models of rickettsial infections have demonstrated that cell-mediated immunity is essential for complete clearance of these pathogens, in particular CD4^+^ T cells [35], although Walker et al. (2001), Feng and Walker (2004), and Ismail and Walker (2005) performed studies to evaluate the roles of T lymphocyte subsets and showed that CD8^+^ T lymphocytes are also crucial for the control and killing of the bacteria resistant to phagocytosis. However, current investigations of T lymphocyte subsets in the peripheral blood of patients with rickettsial infection are scarce [36,37].

The results and data reported by Escárcega-Ávila et al., 2019, in a serological study carried out on veterinary and administrative personnel reported that some of the individuals had been exposed to up to three rickettsial pathogens, *R. rickettsii*, *E. canis*, and *A. phagocytophilum* [38], causing us to hypothesize that there is a possibility that these pathogens are involved in human clinical cases. In this study, we investigated the disruptions in immune parameters in patients who presented a clinical scenario of a single rickettsial or ehrlichial infection and coinfection. Patients admitted to the Hospital Infantil de Especialidades de Chihuahua with clinical manifestations suggesting a rickettsial infection were analysed by PCR to detect and identify the presence of *Rickettsia* spp., *Ehrlichia* spp. or *Anaplasma* spp. Routine protocol laboratory tests were performed, and flow cytometry was used to analyse serum cytokine and chemokine profiles and T-cell subsets. Importantly, we identified the presence of coinfections and ehrlichiosis, and the clinical cohorts were compared by immunological parameters.

## 2. Materials and Methods

### 2.1. Study Subjects and Inclusion Criteria

The study group included fourteen patients (43% female, 57% male) suspected of rickettsial disease from Children’s Hospital of Specialties of the State of Chihuahua, and five healthy controls with ages ranging from 7 to 16 years old, 40% of whom were female and 60% male. Healthy controls were recruited from the state of Chihuahua, Mexico, without a history of a tick bite, autoimmune, cardiovascular, cerebral, and/or osteoarticular disorders, acute infectious processes, or ingestion of drugs before sampling. Every child was punctured in the saphenous vein to obtain blood samples collected in EDTA anticoagulant tubes and tubes with clot activator and gel for serum separation. The patient’s diagnoses required the detection of the gltA gene of *R. rickettsii* and the 16S rRNA gene of *E. canis* by polymerase chain reaction (PCR) using test kits.

### 2.2. Statement of Ethics

The study protocol complied with the guidelines established in the Regulations of the General Health Law in matters of health research, the Declaration of Helsinki, and the Good Clinical Practices issued by the National Bioethics Commission. The Research Ethics Committee of the Faculty of Medicine and Biomedical Sciences of the Autonomous University of Chihuahua also approved it, with registration number CI-056-19.

### 2.3. Haematological and Serum Biochemistry

The blood samples collected were immediately analysed for complete haematological and biochemical profiles at the Hospital Infantil de Especialidades de Chihuahua–Clinical Laboratory. ALB, albumin; ALT, alanine aminotransferase; AP, alkaline phosphatase; AST, aspartate aminotransferase; Ca, calcium; Cl, chloride; CRE, creatinine; ERY, erythrocytes; DB, direct bilirubin; GGT, γ-glutamyltransferase; GLU, glucose; Hb, haemoglobin; HCT, haematocrit; IB, indirect bilirubin; K, potassium; LEU, leukocytes; LYM, lymphocytes; EOS eosinophils; BAS basophils; MON monocytes; MCH, mean corpuscular haemoglobin; MCV, mean corpuscular volume; Mg, magnesium; MPV, mean platelet volume; Na, sodium; NEU, neutrophils; NR, no results; PLT, platelets; TB, and total bilirubin, results are shown in Supplementary Tables S1–S3.

### 2.4. DNA Isolation, PCR Protocol, and Sequencing

The DNA was extracted using a column procedure according to the manufacturer’s instructions, and the DNA was then quantified. The molecular diagnosis was performed using an end-point PCR test as previously described [39,40]. For this purpose, amplification of the 17 kDa protein gene of *Rickettsia* spp., the 16S rRNA ribosomal gene for *Ehrlichia spp*., and *A. phagocytophilum* was performed. Samples positive for the 17 kDa gene were amplified again to amplify the gltA gene to corroborate the genus *R. rickettsii*. In the case of positive samples for *Ehrlichia* spp., nested PCR was performed to identify *E. canis* specifically, further results are shown in Supplementary Figure S1.

### 2.5. Phylogenetic Analysis

Sequences of the selected rickettsial species used were identified by BLAST searches of the nonredundant sequences database of the National Center for Biotechnology Information (NCBI). Amino acid sequences were aligned using the MEGA 11 program [41]. A phylogenetic tree was constructed using the maximum likelihood (ML) method in MEGA software. The reliability of the clustering pattern in the phylogenetic tree was tested by bootstrapping using 1000 pseudosamples [42,43].

### 2.6. Cytokine and Chemokine Profiles

Cytokine and chemokine levels were evaluated from serum samples obtained from patients with rickettsial disease and control donors. For this analysis, the BD cytometric bead array (CBA) human inflammation kit (Catalogue No. 551811), human Th1/Th2/Th17 kit (Catalogue No. 560484), and human chemokine kit (Catalogue No. 552990) were used following the manufacturer’s instructions. Briefly, the reaction was made from a Master mix containing 5 μL of each antibody. The diluent solution was added to a volume per reaction of 50 μL. Then, 50 μL of the test sample and 25 μL of PE were added. The reaction was incubated for 2 h in the dark. Subsequently, 1 mL of wash buffer was added and centrifuged for 5 min at 200× *g*. Finally, 350 μL of wash buffer was added and analysed on an Attune NxT cytometer, in which 10,000 events were recorded at a flow of 100 μL/min. The results were analysed with the FlowJo vX.0.7 program. A graph was generated comparing the size (FWD) and complexity (SSC) of the cells. In this way, the fluorescence of the PE-antibody regions of interest was identified, and the fluorescence intensity corresponding to each cytokine was measured. With these values, the concentration of each cytokine was calculated in picograms per millilitre (pg/mL).

### 2.7. T-Cell Immunophenotype

T-cell subsets were determined using DURAClone Technology (B53328, Beckman Coulter). This panel includes 10 markers in fluorochrome combinations that provide robust population identification, including CD3, CD4, CD8, CD27, CD28, CD45, CD45RA, CD57, CD197 (CCR7), and CD279 (PD-1). Sample preparation and analysis were performed following the manufacturer’s instructions. The expression of CD45RA and CCR7 were analysed within CD4^+^ and CD8^+^ subsets CD45RA, has been shown to discriminate naïve (CD45RA^+^CCR7^+^) and TCM (CD45RA^−^CCR7^+^) from TEM (CD45RA^−^CCR7^−^) and TEMRA^+^(CD45RA^+^CCR7^−^), analysis is described in Supplementary Figure S2. Samples were acquired in the Attune NxT cytometer and analysed in FlowJo software, where the gate strategy to identify the populations was determined (FlowJo version 10; BD Science, San Diego, CA, USA).

### 2.8. Statistical Analysis

Data were recorded in a Microsoft Excel spreadsheet and analysed in the statistical package SAS version 9.0 (SAS Institute Inc., Cary, NC, USA). There were two response variables: (1) the proportion of T lymphocyte and dendritic cell subpopulations, and (2) the concentrations of cytokines, chemokines, and soluble proteins of the inflammatory response, which were dependent on each patient, the clinical findings, and pathogen(s) involved. With these variables, a statistical analysis was performed with the nonparametric Mann—Whitney U and Kruskal—Wallis tests since the data obtained does not comply with the assumptions of normality and homoscedasticity. The Mann—Whitney U test was used to compare the data of the healthy and sick children groups. Follow-up analyses were also performed, and these data were analysed using the Kruskal—Wallis test, which compares the results obtained from the case follow-up samples of the positive patients.

## 3. Results

### 3.1. Overview of Subjects, Molecular Diagnosis, and Detection of Monoinfection and Coinfection

**Figure 1:**
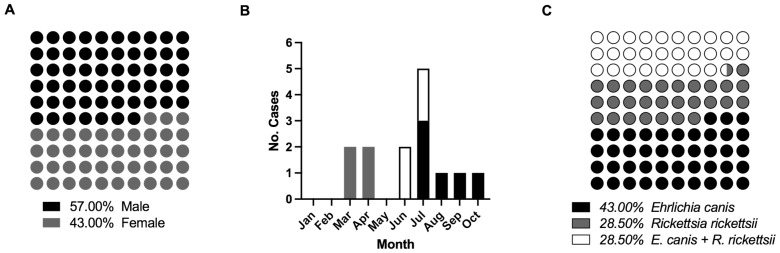
Overview and characteristics from patient samples. (**A**) Gender ratio, (**B**) Case incidence by month for 2021, and (**C**) Ratio of case diseases.

Fourteen blood samples were obtained from children positive for rickettsial disease from January to December 2021. The sex ratio of males to females was eight (57%) to six (43%), respectively. The ages of the individuals varied between one and 15 years, with an average of six years (Figure 1A). Six (43%) samples were acquired during March and June, five (35%) in July, and the last three (22%) samples were acquired from August to October (one sample each month), Figure 1B. All the samples were analysed by PCR and four (28.5%) patients were infected with *R. rickettsii*, six (43%) with *E. canis*, and four (28.5%) with both bacteria in coinfection by yielding amplicons of the expected size for each pathogen and DNA sequencing confirmation (Figure 1C and Supplementary Figure S1), characteristics of primers used are described on Table 1. None of the samples were positive for *A. phagocytophilum*. This report may be the first description of *E. canis* mono-infection and its participation in coinfection with *R. rickettsii* in pediatric patients.

Patients admitted to the hospital displayed an average of 5.35 days of the course of the disease, and essential to highlighting the bias provided by the parents, data is obtained at the moment of admission, not necessarily represent the accurate day of the development of the disease (Table 2). Of the fourteen patients, three were admitted to general hospital wards (21.4%) and eleven to the intensive care unit (78.5%). Ten patients required chronotropic meds (support with amines). Five patients presented some neurological complications, such as seizures, encephalopathy, and neuropathy, and required the use of anticonvulsant management and subsequent follow-up by neurology and rehabilitation even after discharge (Table 2). All diseases display the same clinical picture, according to the days of evolution, therefore, should be no difference between the management of one disease or the other, or the co-infection. For the time being, Ehrlichiosis should be given the same importance as Rickettsiosis, as it presents, at least in the patients analyzed, the same degree of severity as Rickettsiosis.

### 3.2. Phylogenetic Analysis

**Figure 2:**
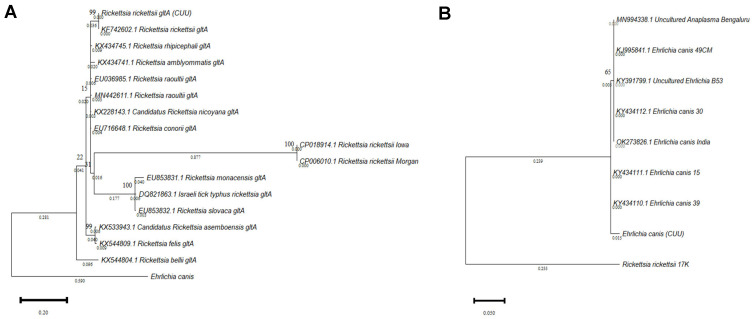
Phylogenetic analysis of sequences obtained from nucleic acids isolated from patient samples. (**A**) Molecular phylogenetic analysis of the *Rickettsia* gltA gene using the maximum likelihood (ML) method generated with the 3-parameter Tamura Model with 1000 bootstrap replicates. (**B**) *Ehrlichia* molecular phylogenetic analysis of the 16S rRNA gene using the maximum likelihood (ML) method generated with the Kimura 2-parameter model with 1000 bootstrap replicates. Both trees with the highest logarithmic probability are shown. The number of trees in which the associated taxa clustered is shown above the branches. The tree is drawn to scale, with branch lengths below. Each sequence is indicated by its GenBank accession number.

The results showed the efficacy of the 16S and gltA molecular markers in identifying *R. rickettsii* and *E. canis* species. Therefore, these markers are adequate for the phylogenetic discrimination of tick-borne diseases in the State of Chihuahua, and could be considered as a pipeline analysis for correctly identifying these pathogens. According to the Bayesian Information Criterion (BIC) score, the best model for the sequences was evaluated based on all the likelihoods. Two phylogenetic trees were generated, one for *R. rickettsii* gltA with the Tamura 3-parameter model (Figure 2A) and the *E. canis* tree (Figure 2B) built with the Kimura 2-parameter model. In both cases, bootstrap values of 1000 bootstrap replicates were used.

### 3.3. Laboratory Panel Tests

Haematologic and biochemical profiles were recorded from 11 of 14 children’s samples (Table 3). For liver enzymes, such as GGT, FA, ALT, AST, and coagulation times (thromboplastin and prothrombin), we found elevated ranges in 100% of the children in the study. Sixty-four percent of the patients had leukocytosis due to neutrophilia, mainly in positive samples for *R. rickettsii*. Fifty-five percent of the children showed alterations such as hypoalbuminemia, hypoproteinemia, lymphopenia, and thrombocytopenia, and normochromic normocytic anaemia was observed in forty-five percent of the children. The coinfected group seemed to be the most affected, exhibiting the most haematologic and biochemical alterations.

### 3.4. Cytokine Profiles

**Figure 3:**
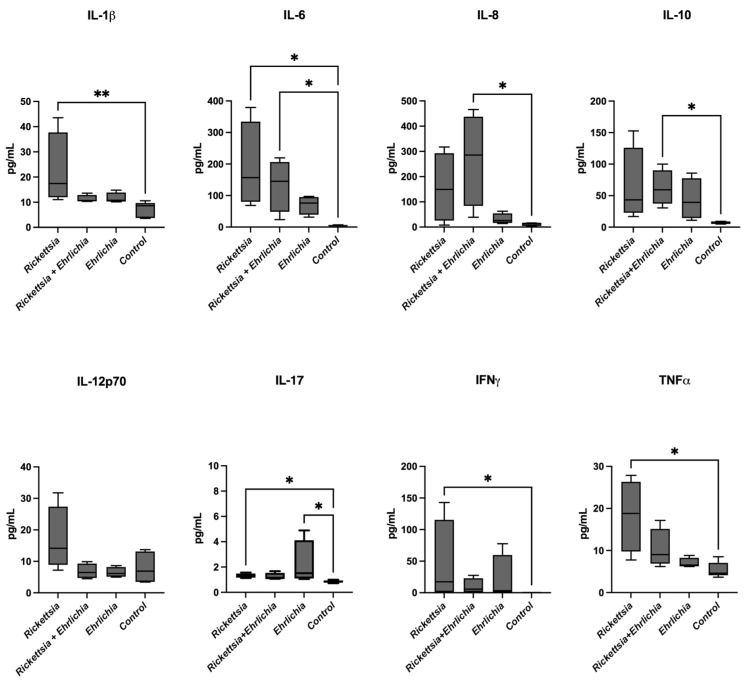
Cytokine levels in sera from children with rickettsiosis, ehrlichiosis, coinfection (rickettsiosis and ehrlichiosis), and control. Samples were analysed in parallel using the bead-based human inflammatory cytokine kit assay. Data are expressed as the mean ± SD. Statistical analyses were performed using the Kruskal—Wallis multiple comparison test. Statistically significant differences are displayed on top of each plot (* *p* < 0.05, ** *p* < 0.01).

The cytokines IL-1β, IL-6, IL-8, IL-10, IL-12p70, IL-17, IFNγ, and TNFα were analysed and compared among the four groups. Significant differences were found in IL-1β upregulated expression between rickettsial patients and control donors. A tendency towards increased expression was observed in all groups in contrast with the control. The inflammatory cytokine IL-6 displayed significant differences in the *Rickettsia*- and *Ehrlichia*-infected groups, in contrast with controls. On the other hand, IL-8 and IL-10 cytokines were significantly different between the control and coinfection groups. For IL-12p70, no significant differences were observed; however, a slight increase in IL-17 was observed in the *Rickettsia*- and *Ehrlichia*-positive groups in contrast with the control group. Additionally, IFNγ and TNFα showed significantly increased expression levels between *Rickettsia* and the control group, highlighting the upregulation pattern in TNF expression. The results obtained from this cytokine screening suggest a severe inflammatory scenario among the interrogated groups, with particular emphasis in *Rickettsia rickettsii* patient group. These data are shown in Figure 3.

### 3.5. Chemokine Profiles

**Figure 4:**
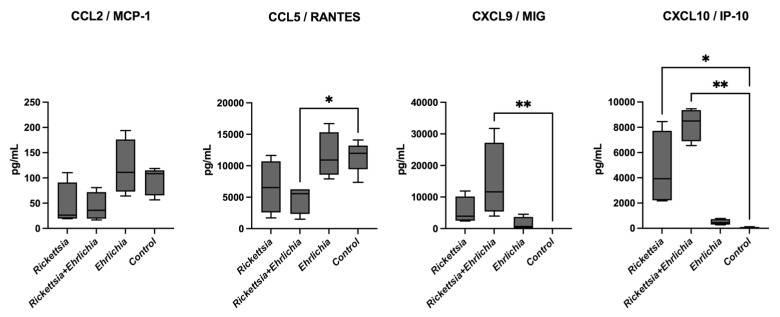
Chemokine levels in sera from children with rickettsiosis, ehrlichiosis, coinfection (rickettsiosis and ehrlichiosis), and control. Samples were analysed in parallel using the bead-based human inflammatory cytokine kit assay. Data are expressed as the mean ± SD. Statistical analyses were performed using the Kruskal—Wallis multiple comparison test. Statistically significant differences are displayed on top of each plot (* *p* <0.05, ** *p* <0.01).

The chemokines CCL2/MCP-1, CCL5/RANTES, CXCL9/MIG, and CXCL10/IP-10 were analysed and compared among the four groups. After data analysis, the chemokine CCL2/MCP-1 showed no difference in expression between the control and *Ehrlichia*-positive groups and a trend towards downregulation in the *Rickettsia*- and coinfected groups. In CCL5/RANTES, no significant differences were observed among the control group compared with the *Rickettsia* and *Ehrlichia* groups; however, significantly downregulated expression was observed in the coinfection group. For CXCL9/MIG, a significant increase was detected within the coinfection and control panels along with CXCL10/IP-10, and significant differences were found in *Rickettsia* and coinfected panels when compared with the control and *Ehrlichia* panels. In contrast with the cytokine profile, on the scrutinized chemokines CCL5/RANTES, CXCL9/MIG, and CXCL10/IP-10 significant differences were observed in the coinfection group, evoking a T cell migration, homing, and recruit signal pattern. These data are shown in Figure 4.

### 3.6. Immunophenotyping CD4^+^ T Cells

**Figure 5:**
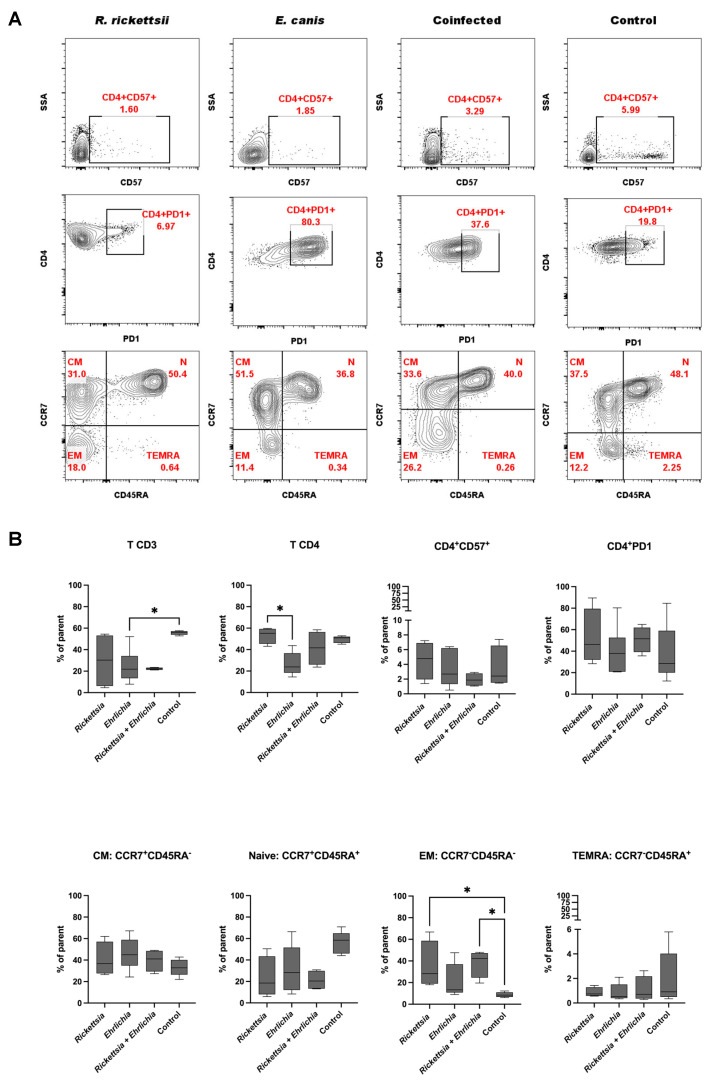
CD4^+^ T-cell phenotype analysis from children with rickettsiosis, ehrlichiosis, coinfection (rickettsiosis and ehrlichiosis), and control. (**A**) Representative plots of a T-lymphocyte immunophenotype. Cells are first gated on total lymphocytes (FSC/SSC and/or SSC/CD45). Populations of each lymphocyte subset are then shown in a plot and quadrant gating provides the percentage of lymphocytes possessing each cell marker (e.g. the percentage of CD45^+^/CD3^+^/CD4^+^/CD57^+^ lymphocytes). Naïve (CCR7^+^ CD45RA^+^), central memory (CM; CCR7^+^ CD45RA^−^), effector memory (EM; CCR7^−^ CD45RA^−^) and TEMRA (CCR7^−^ CD45RA^+^) T cells. (**B**) CD4 T cell populations expressed as a percentage of gated lymphocytes. Data are expressed as the mean ± SD. Statistical analyses were performed using the Kruskal—Wallis multiple comparison test. Statistically significant differences are displayed on top of each plot (* *p* <0.05).

The analysis of the T-cell immunophenotype experiments showed a lower percentage of the CD3^+^ T-cell population compared to the control group, despite the significant difference in the coinfection group. In terms of the CD4^+^ T subpopulation cells, nonsignificant differences were observed; however, a significant reduction was observed between the *Rickettsia* and *Ehrlichia* groups. Naïve CD4^+^CD45RA^+^CCR7^+^ T cells tended to be reduced compared to the control group (no statistical significance). On the other hand, central memory (CM) cells and TEMRA cells showed not significant differences. Finally, in the effector memory (EM) population, we detected an increase in the *Rickettsia* and coinfection groups compared to the control group (Figure 5A,B). This data may suggest the role of the EM population in providing protective immunity against pathogens. This is consistent with the lower proportion of the naïve CD4^+^ T cell population.

### 3.7. Immunophenotyping CD8^+^ T Cells

In the case of the CD8^+^ T population, the *Rickettsia* infection group displayed a significantly lower proportion of cells in contrast with the control, *Ehrlichia*, and coinfection panels.

**Figure 6:**
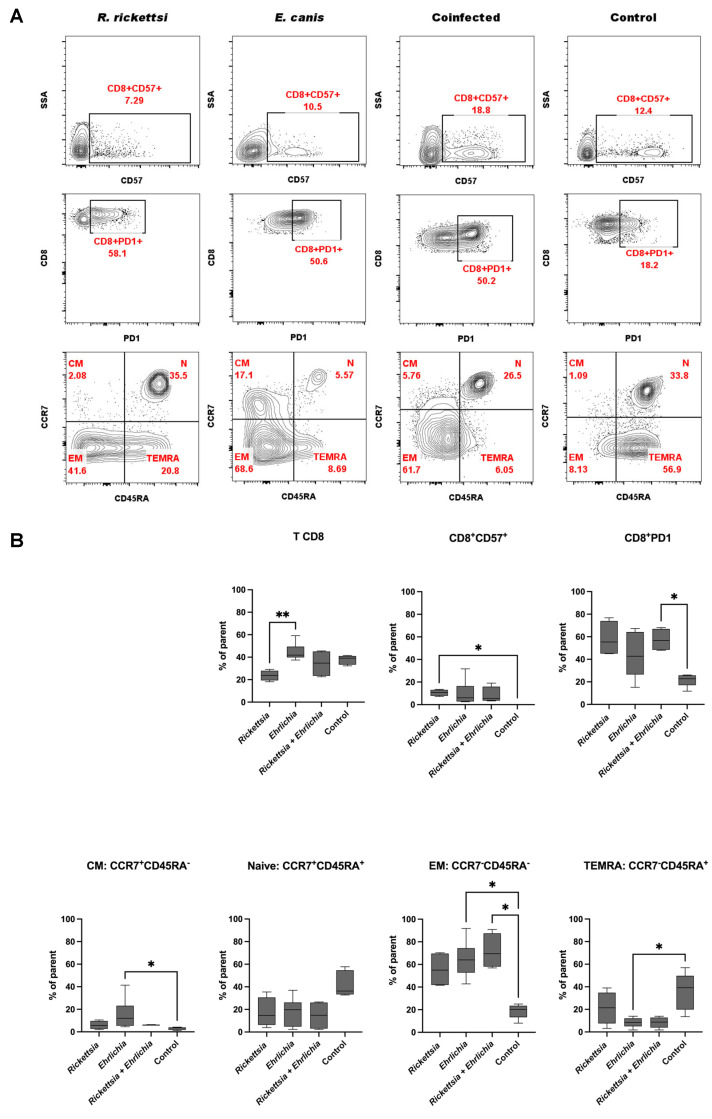
CD8^+^ T-cell phenotype analysis in blood obtained from children with rickettsiosis, ehrlichiosis, coinfection (rickettsiosis and ehrlichiosis), and control. (**A**) Representative plots of a T-lymphocyte immunophenotype. Cells are first gated on total lymphocytes (FSC/SSC and/or SSC/CD45). Populations of each lymphocyte subset are then shown in a plot and quadrant gating provides the percentage of lymphocytes possessing each cell marker (e.g. the percentage of CD45^+^/CD3^+^/ CD8^+^/CD57^+^ lymphocytes). Naïve (CCR7^+^ CD45RA^+^), central memory (CM; CCR7^+^ CD45RA^−^), effector memory (EM; CCR7^−^ CD45RA^−^) and TEMRA (CCR7^−^ CD45RA^+^) T cells. (**B**) CD8 T cell populations expressed as a percentage of gated lymphocytes. Data are expressed as the mean ± SD. Statistical analyses were performed using the Kruskal—Wallis multiple comparison test. Statistically significant differences are displayed on top of each plot (* *p* <0.05, ** *p* <0.01).

For CD8^+^CD57^+^ T lymphocytes, the result was significant only between the *Rickettsia* group and the control. However, a slight increase in this population was observed in all groups analysed. In contrast, the CD8^+^PD1^+^ cell population showed significant changes for the coinfected group compared to the control group, and also showed increased levels in comparison to the no infection group. This result suggests exhausted cells, which may come from prolongated exposition to the pathogen, and persistent antigen exposure under not optimal conditions. In the case of the CD8^+^CCR7^+^CD45RA^−^ (CM) population, only a moderately significant increase was detected in the *Ehrlichia* group with respect to the control. In the CD8^+^CCR7^+^CD45^+^ (Naïve) cell population, no significant differences were found. For the cell population CD8^+^CCR7^−^CD45RA^−^ (EM), a moderately significant increase in the *Ehrlichia* and coinfected groups was observed compared to the control, but no significant changes were observed in the other groups. Results are consistent with the cell response; the increase is related to the pathogen encounter. These cells could play a role in providing immunity against pathogens. Finally, CD8^+^CCR7^−^CD45^+^ (TEMRA) cells tended to be decreased compared to the control, but was only significant in the group of patients infected with *Ehrlichia* (Figure 6A,B).

In contrast to CD4^+^ T cells, the CD8^+^ T cells populations are more susceptible to *Ehrlichia canis* and coinfection.

## 4. Discussion

This manuscript may be the first report worldwide of *E. canis* and its association in coinfection with *R. rickettsii* in paediatric patients. Blood samples were obtained from children suspected of rickettsial infection and were analysed in 2021. All children positive for these pathogens showed an array of haematological and biochemical clinical alterations. In this study, we worked with three different groups of patients with rickettsial diseases. We compared the clinical presentation of infected patients and determined the cytokine and chemokine profile in each group and the T-cell phenotype and compared them between groups with the respective controls.

In contrast to the USA, the northwestern region of Mexico is an endemic zone to *R. sanguineus*, a monotropic vector of dogs, which usually feeds on humans when temperatures are high, and this species is a competent vector of *R. rickettsii* [44], *A. phagocytophilum* [45], *E. canis*, and *A. platys* [46]. However, the transmission of other intracellular pathogens [47], *Hepatozoon canis* [48] among others, and their participation in coinfection with these rickettsial diseases is not excluded.

In agreement with other studies, most of the cases in northern Mexico occur from spring to autumn, with a greater incidence in the summer season, when *R. sanguineus* has high reproductive activity [49]. Climate-prevailing factors in Chihuahua may be related to the presentation of clinical cases; it is known that at higher temperatures, *R. sanguineus* has an aggressive feeding behaviour, increasing the risk for bites in humans [50]. Higher population densities of dogs and *R. sanguineus* have been established as risk factors for acquiring rickettsial diseases in northern Mexico, as has been previously suggested by Escárcega Ávila et al. in a study performed in Juarez City in 2018, in which they reported a prevalence in dogs of 43%, 40%, and 28% of *R. rickettsia*, *E. canis*, and *A. phagocytophilum,* respectively.

Patients with rickettsial diseases display an unspecific fever syndrome with characteristic temperatures up to 40 °C, different clinical symptoms, and several haematological and biochemical alterations. Likewise, for the patient cohort described in this manuscript, the total rickettsiosis, ehrlichiosis, and coinfection cases are consistent with previous reports that describe an increase in hepatic enzymes (ALT, AST, and AP), clinical chemical analytes with alterations, such as an increased clotting time (CT), prothrombin time (PT), and partial thromboplastin time (PTT). These results are congruent with the data reported by Delgado-De la Mora et al., 2018 [51].

Previous studies have shown that up to 90% of patients with any of these inflammatory diseases present with thrombocytopenia, and in some studies, it is related to the severity of the clinical status in paediatric patients [21,51]. Normochromic normocytic anaemia and hypoalbuminemia are other alterations similar to those reported by the Centers for Disease Control and Prevention [20,52]. These alterations are caused by blood loss due to the destruction of the endothelium, damage to liver tissue and physiological compensatory mechanisms. It is fundamental to consider that the presentation of the clinical status and the alterations observed in patients infected with these bacteria will depend on intrinsic host factors, such as immune performance, age, and nutritional status, among others [53].

Clinical studies have reported *E. canis* infection in adults Perez et al., 2006 and detected a prevalence of 30% in patients suspected of rickettsial disease in Venezuela. Silva et al., 2014 reported an isolated case in Oaxaca City from a dog groomer with a subclinical presentation of this illness. Several clinical studies have reported Ehrlichiosis caused by *Ehrlichia chaffeensis* around the world. In Mexico, the first registered case of this pathogen was from a 35-year-old man in Mexico State, and the most recently reported case was a homeless man in 2020 who lived in close proximity to dogs [54,55,56].

We observed statistically significant differences for IL-8, RANTES, CXCL9/MIG, and CXCL10/IP-10 chemokines when comparing the coinfected patients and control group, and IP-10 was significant for patients infected by *R. rickettsii* compared to the control group. In cytokine secretion, significant differences were observed for IL-1β, IL-6, IL-17, IFNγ, and TNF-α among the *R. rickettsii* positive group compared to the control group. The coinfected group displayed altered levels of IL-6, IL-8, and IL-10 compared to the control group. Finally, significant differences were observed in all of the subpopulations of CD8^+^ T lymphocytes when we compared the group positive for *R. rickettsii* and the group positive with *E. canis* with controls.

A few cases of coinfections with rickettsial diseases have been reported in Mexico, as reported by Licona-Enríquez et al., 2018, where dengue virus and *R. rickettsii* were diagnosed and responsible for the death of a woman in Sonora State. Dzul-Rosado et al., 2021 reported a clinical case in a child with *R. rickettsii* coinfected with *Leptospira* spp. in Yucatán, with a fatal outcome. In a retrospective study of HIV patients, Paddock et al., 2001 found *E. chaffeensis* and *Ehrlichia ewingii* coinfecting the hosts located in states belonging to the tick belt in the southeastern region of the United States. Raczniak et al., 2014 described a fatality case in a Native American coinfected with *R. rickettsii* and *Streptococcus pyogenes* in Arizona. Finally, in North Carolina, Carpenter et al., 1999 performed a two-year study in which they found patients who presented with *R. rickettsii* in coinfection with *E. chaffeensis* [22,51,57,58,59].

The data obtained in this study are in agreement with data obtained by Rauch et al., 2018, who showed that *R. felis*-positive children display a significant difference in the serum levels of IP-10, MCP-1, and IL-8 in a patient cohort compared to the control group. For these cytokines, the authors reported no significant differences in patients coinfected with P. falciparum compared to the positive *R. rickettsii* group [60].

In contrast to the results obtained by Rauch et al., 2018 in our study, we observed a significant difference between the coinfected and *R. rickettsii* monoinfected groups. This could be a result of the aforementioned, in which in some cases of coinfection the mechanism of action of the pathogens involved boost or act synergistically, increasing the extent of the injury, severity, and complexity of the clinical status [60].

In addition, Clifton et al., 2005, found an increase in the chemokines IL-8 and MCP-1 by measuring mRNA expression in vascular endothelial cells cultured with *R. rickettsii.*

Experiments performed by Rydkina et al., 2005, 2007 in human vascular endothelial cells and umbilical cord cells infected with *R. conorii*, *R. tiphy*, and *R. rickettsii* showed an upregulation of IL-8, MCP-1, IP-10, MCP-2, and RANTES expression [61,62]. Bechah et al., 2008 observed upregulated mRNA expression of TNFα, IL1α, IL-6, and CXCL-10 and downregulated expression of CCL-2 and CCL-5 after infecting a line of murine lung microvascular endothelial cells with *R. prowazekii*, thus associating the expression of cytokines in damaged cells with bacterial virulence [63,64]. The cell endothelium injury caused by these bacteria activates a canonical proinflammatory response triggering the expression of cytokines, such as IL-1β, TNFα, IFNγ, and IL-6, as well as an inhibitory stimulus to control this response through IL-10.

The inflammatory response stimulated by the pathogenic action of rickettsial bacteria triggers immune mechanisms for infection control dependent on cellular immunity and CD4 and CD8 T lymphocytes [64,65] In this study, we report the first findings on inflammatory responses during the acute phase of these infections. Significant differences were observed for the IL-8, RANTES, CXCL9/MIG, and CXCL10/IP-10 chemokines among the coinfected group and the control group. For IP-10, the expression in the *Rickettsia* group was significantly different compared to the control (Figure 3).

## 5. Conclusions

The main limitation of this manuscript is the number of patients included in the study was low. However, they are the total number of cases that occurred during the study period. Comparison of the immune response between age ranges is a question to be addressed, with the precedent knowledge, relating to the tolerogenic response against infection in paediatric patients. Further experiments should characterize other cell lineages, such as NK cells, dendritic cells, monocytes, and neutrophils, and expand the panel of cytokines. Additionally, it is essential to address the problem with other types of technologies in a synchronous conjugation of single-cell gene analysis that will provide a more detailed picture of the disruption caused by these pathogens in different cells of the immune system and thus explore the presence of other pathogens that were left out of the simple molecular screening used in this study.

## Figures and Tables

**Table 1:** Summary of primers used in this work and their characteristics.

Bacteria	Target Gene	Primer Sequence	Amplicon Size bp.
*Ehrlichia* spp.	16S rRNA	F-EHR16SD GGTACCYACAGAAGAAGTCC R-EHR16SR TAGCACTCATCGTTTACAGC	345
*R. rickettsii*	17KDa	F-17K178 GGTGCATTACTTGGAGCAG R-17K452 GGTTGGCGGCATGCATTAC	274
*A. phagocytophilum*	16S rRNA	F-Phago GGCATGTAGGCGGTTTTCGGTAAGTT R-Phago CCCCACATTCAGCACTCATCGTTTA	262
*R. rickettsii*	glta	F-Cs78 GCAAGTATCGGTGAGGATGTAAT R-Cs323 GCTTCCTTAAAATTCAATAAATCAGGAT	401
*Nested PCR* *Ehrlichia* spp.	16S rRNA	F-ECC AGAACGAACGCTGGCGGCAAGC R-ECB CGTATTACCGCGGCTGCTGGCA Nested F-ECAN CAATTATTTATAGCCTCTGGCTATAGGA R-HE3 TATAGGTACCGTCATTATCTTCCCTAT	483 & 387

**Table 2:** Main characteristics of the study population.

Characteristic	*Rickettsia* (N = 4)	Coinfection *Rickettsia + Ehrlichia* (N = 4)	*Ehrlichia* (N = 6)	Control (N = 5)
**Age**	4 ± 13	2 ± 13	1 ± 7	7 ± 13
**Male sex–No. (%)**	1 (75)	2 (50)	2 (33.3)	2 (40)
**Course of disease days–Avg.**	3 to 9 (3)	4 to 5 (2.25)	2 to 7 (1.5)	N.D.
**Days on ICU–Avg.**	3 to 7(2.5)	0 to 18 (4.5)	0 to 12 (2)	N.D.
**Days on the wards**	5 to 7	3 to 16	0 to 7	N.D.
**Chronotropic** **Received/Total**	3/4	3/4	2/6	N.D.
**Disease impact/Total**	2 ^a^/4	1 ^b^/4	1 ^a^/1 ^c^/6	N.D.

^a^ Encephalopathy; ^b^ Neuropathy and ^c^ Vasculitis.

**Table 3:** Summary and comparison of laboratory panel tests.

Haematologic Alteration	*R. rickettsii*	*E. canis*	Coinfected	Total	%
ELE	4	3	4	11	100
Elevated coagulation times	4	3	4	11	100
Leucocytosis by neutrophilia	3	1	2	7	64
Thrombocytopenia	1	1	4	6	55
Lymphopenia	0	2	4	6	55
Hypoalbuminemia	2	0	4	6	55
Hypoproteinemia	2	0	4	6	55
NNA	3	1	1	5	45

NNA: normocytic normochromic anaemia, ELE: elevated liver enzymes.

## Data Availability

Additional data is available on supplementary materials section.

## Supplementary Materials

The following supporting information can be downloaded at: https://www.mdpi.com/article/10.3390/pathogens11111351/s1, Figure S1: Agarose gels at 1.8% stained with Ethidium Bromide; Figure S2: Gate strategy analysis; Table S1: Laboratory cabinet tests summary and comparison; Table S2: Laboratory cabinet tests Biochemistry parameters; Table S3: Laboratory cabinet tests electrolytes parameters.
