# Supplementary material for: Immune Monitoring of Paediatric Patients Infected with Rickettsia rickettsii, Ehrlichia canis and Coinfected
Source: Pathogens. 2022 Nov 15;11(11):1351. doi: 10.3390/pathogens11111351 (PMC9696171; doi:10.3390/pathogens11111351)
Supplement: Supplementary file 1 [file pathogens-11-01351-s001.zip › pathogens-1949131-supplementary.pdf]

Supplementary Materials

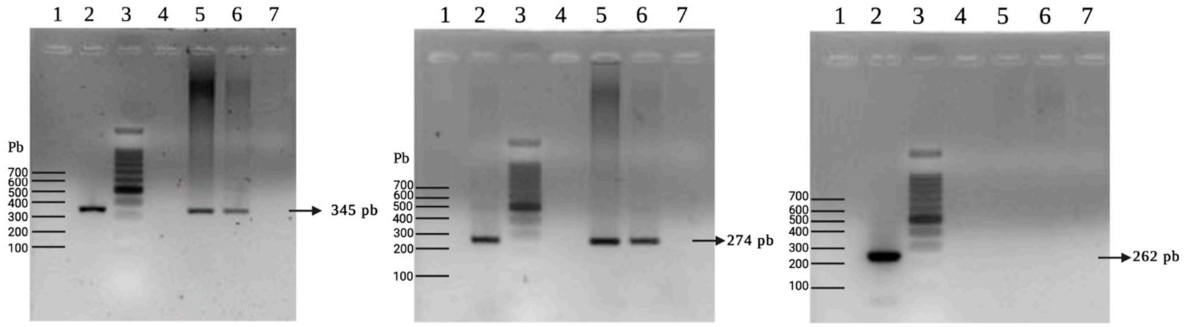

(**A**) (**B**) (**C**)

Supplementary Figure S1. Agarose gels at 1.8% stained with Ethidium Bromide. (**A**) *Ehrlichia spp* (**B**) *R. rickettsii* (**C**) *A. phagocytophilum* Lanes: (1) empty, (2) Positive control, (3) Promega MR 100pb molecular weight marker, 4) Negative control, (5) Experimental sample, (6) Experimental sample, (7) Experimental sample. Expected Amplicon sizes 345, 274 and 262 bp for *E. canis, R. rickettsii* and *A. phagocytophilum* respectively

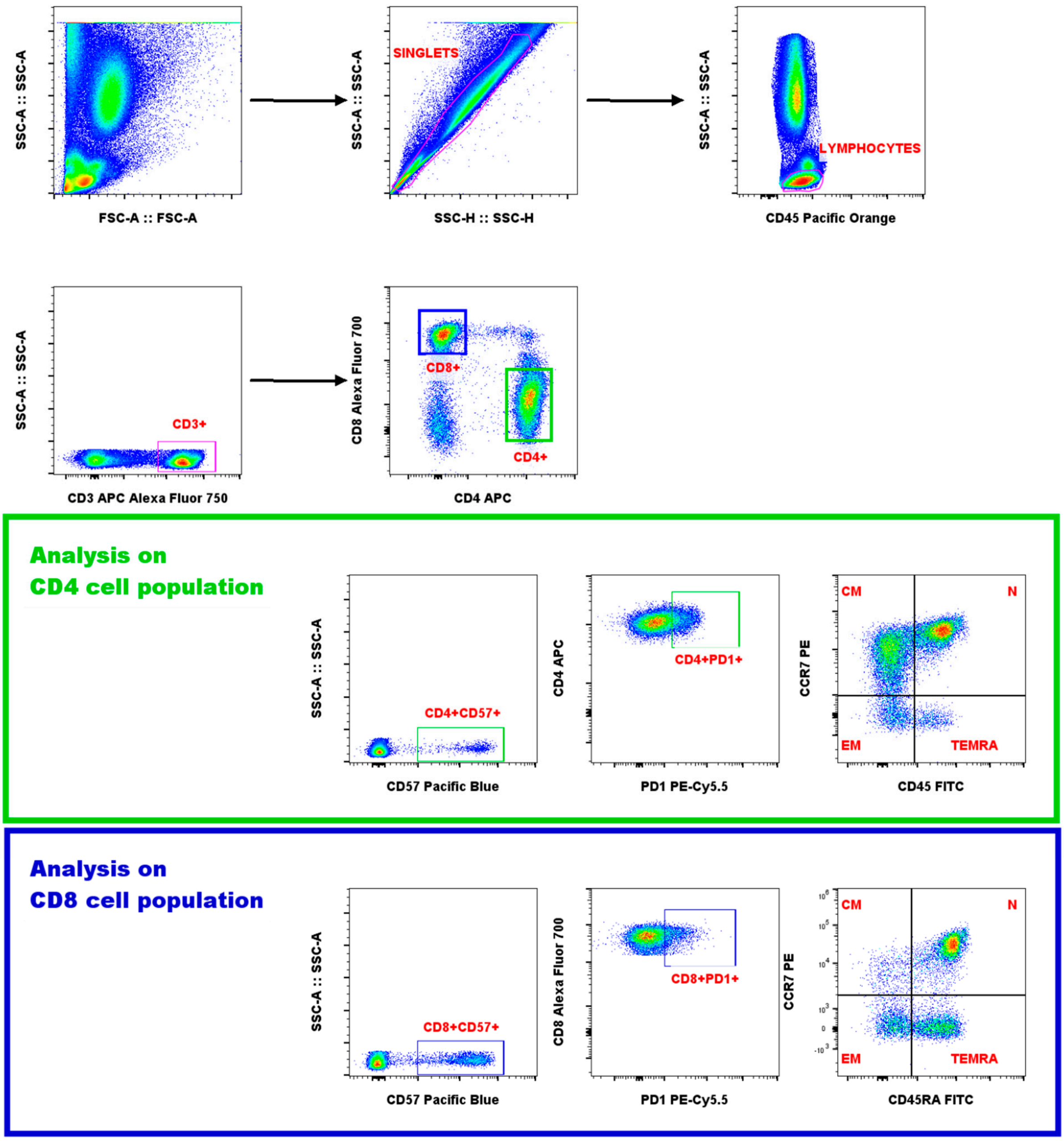

Supplementary Figure S2. Gate strategy analysis. The expression of CD45RA and CCR7 were analyzed within CD4^{+} and CD8^{+} subsets CD45RA, has been shown to discriminate naïve (CD45RA^{+}CCR7^{+}) and TCM (CD45RA^{-}CCR7^{+}) from TEM (CD45RA^{-}CCR7^{-}) and TEMRA^{+}(CD45RA^{+}CCR7^{-}).

Supplementary Table S1. Laboratory cabinet tests summary and comparison

Patient	Age	Gender	Date of reception	LEU	NEU	LYM	MON	EOS	BAS	ERY	Hb	HCT	MCV	MCH	MCHC	PLT	MPV
1	7	F	03/11/21	11.52	7.70	2.53	0.27	0	0.14	4.17	11.6	32.5	78	27.7	35.5	19	13.3
2	4	F	03/25/21	14.39	10.27	2.66	0.98	0	0.1	4.5	12.5	36	78.8	27.4	34.8	236	7.2
3	5	F	04/04/21	10.91	8.62	1.51	0.29	0	0.07	4.18	11.8	34	81.3	28.3	34.9	150	8.6
4	3	M	04/16/21	8.5	6.03	1.93	0.24	0.04	0.05	4.07	11.3	32.7	80.5	27.8	34.5	157	6.8
5	7	F	06/08/21	3.7	1.7	1.47	0.27	0.08	0.04	3.9	11.6	35.6	90.4	29.4	32.5	185	7.4
6	3	M	07/21/21	5.65	4.28	0.91	0.23	0.01	0.03	4.63	12.5	36	77.8	27	34.7	153	7.9
7	5	M	06/21/21	18.38	16.36	1	0.81	0.02	0.03	4.7	13.1	39	82.3	27.7	33.7	429	6.8
8	2	F	07/06/21	5.38	3.76	1	0.23	0.01	0.05	3.9	10.8	33.3	84	27.2	32.4	36	12.9
9	13	M	07/06/21	20.7	17.8	0.94	0.57	0.01	0.23	4.83	14	42.4	87.7	29	33.1	15	15.1
10	10	F	07/08/21	3.06	2.7	0.15	0.09	0	0.01	4.2	12.8	38.5	91.1	30.2	33.2	78	10.4
11	10	M	07/13/21	24.4	21.53	1.16	0.9	0.01	0.16	4.43	13	38.4	86.7	29.3	33.7	53	14.3

Supplementary Table S2. Laboratory cabinet tests Biochemistry parameters

Patient	Age	Gender	Date of reception	TB	IB	DB	ALB	GGT	AP	ALT	AST	GLB	CRE	GLU
1	7	F	03/11/21	1.1	0.4	0.6	2.1	84	165	98	184	2	0.4	93
2	4	F	03/25/21	0.5	0.3	0.2	4.5	19	239	25	46	3	NR	NR
3	5	F	04/04/21	0.5	0.3	0.3	3.1	136	264	100	127	3	0.2	78
4	3	M	04/16/21	0.5	0.4	0.1	4	22	157	22	72	3	0.3	96
5	7	F	06/08/21	0.1	0	0	3.2	14	120	15	32	2	0.5	85
6	3	M	07/21/21	0.3	0.3	0.1	3.8	18	127	78	186	3	0.4	102
7	5	M	06/21/21	0.3	0	0.3	4.9	15	221	21	38	3	0.3	61
8	2	F	07/06/21	0.5	0.2	0.2	2.8	30	151	33	104	2	0.2	71
9	13	M	07/06/21	6.1	0.8	5.3	2.6	74	215	68	302	2	3.5	90
10	10	F	07/08/21	3.1	0.4	2.7	2.9	95	242	112	252	3	0.5	113
11	10	M	07/13/21	7.3	0.7	6.6	2.8	522	454	119	243	3	1.6	109

Supplementary Table S3. Laboratory cabinet tests electrolytes parameters

Patient	Age	Gender	Date of reception	K+	Na+	Cl-	Ca2+	Mg2+	IP
1	7	F	03/11/21	120	4.8	99	7.3	2	2.8
2	4	F	03/25/21	132	3.9	102	8.9	1.8	3
3	5	F	04/04/21	137	4.5	103	8.2	1.9	3.9
4	3	M	04/16/21	130	4.6	97	7.8	2.4	4.2
5	7	F	06/08/21	140	5.2	117	8.7		4.6
6	3	M	07/21/21	129	4.3	96	9.1	1.8	2.8
7	5	M	06/21/21	141	3.3	106	9.9	2.1	5.4
8	2	F	07/06/21	129	3.6	98	8.5	2.2	3.5
9	13	M	07/06/21	120	5.2	80	7.5	3	5.6
10	10	F	07/08/21	129	4.1	99	7.6	2.6	2.9
11	10	M	07/13/21	137	3.8	100	8.3	2.8	4.8

Abreviations

ALB, albumin; ALT, alanine aminotransferase; AP, alkaline phosphatase; AST, aspartate aminotransferase; Ca, calcium; Cl, chloride; CRE, creatinine; ERY, erythrocytes; DB, direct bilirubin; GGT, γ-glutamyltransferase; GLU, glucose; Hb, hemoglobin; HCT, hematocrit; IB, indirect bilirubin; K, potassium; LEU, leukocytes; LYM, lymphocytes; EOS eosinophils; BAS basophils; MON monocytes; MCH, mean corpuscular hemoglobin; MCV, mean corpuscular volume; Mg, magnesium; MPV, mean platelet volume; Na, sodium; NEU, neutrophils; NR, no results; PLT, platelets; TB, total bilirubin.
